# Identification and Sequence Analysis of a Novel Ilarvirus Infecting Sweet Cherry

**DOI:** 10.3390/plants10030514

**Published:** 2021-03-10

**Authors:** Chrysoula G. Orfanidou, Fei Xing, Jun Zhou, Shifang Li, Nikolaos I. Katis, Varvara I. Maliogka

**Affiliations:** 1 Laboratory of Plant Pathology, Faculty of Agriculture, Forestry and Natural Environment, School of Agriculture, Aristotle University of Thessaloniki, 54124 Thessaloniki, Greece; chorfani@agro.auth.gr (C.G.O.); katis@agro.auth.gr (N.I.K.); 2 State Key Laboratory of Biology of Plant Diseases and Insect Pests, Institute of Plant Protection, Chinese Academy of Agricultural Sciences, Beijing 100193, China; xingfly1218@163.com (F.X.); junzhou2019@aliyun.com (J.Z.); lishifang2003@aliyun.com (S.L.)

**Keywords:** ilarvirus, novel species, *Prunus*, high-throughput sequencing

## Abstract

In the present study, we utilized high throughput and Sanger sequencing to determine the complete nucleotide sequence of a putative new ilarvirus species infecting sweet cherry, tentatively named prunus virus I (PrVI). The genome of PrVI is comprised of three RNA segments of 3474 nt (RNA1), 2911 nt (RNA2), and 2231 nt (RNA3) and features conserved motifs representative of the genus *Ilarvirus*. BlastN analysis revealed 68.1–71.9% nt identity of PrVI with strawberry necrotic shock virus (SNSV). In subsequent phylogenetic analysis, PrVI was grouped together with SNSV and blackberry chlorotic ringspot virus (BCRV), both members of subgroup 1 of ilarviruses. In addition, mini-scale surveys in stone fruit orchards revealed the presence of PrVI in a limited number of sweet cherries and in one peach tree. Overall, our data suggest that PrVI is a novel species of the genus *Ilarvirus* and it consists the fifth member of the genus that is currently known to infect *Prunus* spp.

## 1. Introduction

*Prunus* spp. are infected by a significant number of plant viruses, including at least four members of the genus *Ilarvirus* (family *Bromoviridae*): apple mosaic virus (ApMV), American plum line pattern virus (APLPV), prune dwarf virus (PDV), and prunus necrotic ringspot virus (PNRSV). Among these viruses, PDV and PNRSV are the most prevalent and have been associated with various stone fruit diseases, such as “ringspot disease” or “peach stunt disease” [1]. Nevertheless, most ilarviruses cause latent infections in their *Prunus* hosts [1], thus their contribution to disease development is a difficult field to explore. Ilarviruses have four to five open reading frames (ORFs) encoded by their tripartite, positive-sense, single-stranded RNA genome. RNA-1 and RNA-2 encode proteins associated with replication, whereas RNA-3 harbors ORFs that encode the movement (MP) (5′ terminal) and the coat protein (CP) (3′ terminal). Based on serological and molecular data, ilarviruses are clustered into four subgroups, of which subgroups 1 and 2 encode an additional protein, named 2b (3′ terminal of RNA-2), putatively associated with silencing suppression activity [2,3].

In a four-year (2009–2013) survey, conducted on sweet cherry (*Prunus avium*) orchards in Imathia region in Northern Greece for the presence of Betaflexiviridae viruses [4], a sample was collected from a symptomless sweet cherry tree (cv. Ferrovia, coded ‘c18′). The sample was subjected to high throughput sequencing (HTS) analysis, which revealed among other known sweet cherry viral pathogens the presence of a putative new ilarvirus species. Based on the HTS data and further Sanger sequencing analysis the complete genome of the novel ilarvirus species was determined for which the putative name ‘prunus virus I (PrVI)’ is proposed. Phylogenetic relationships and sequence comparison with other characterized ilarviruses support the designation of PrVI as a novel species in the genus *Ilarvirus*. Preliminary surveys in Imathia region were also conducted, in order to investigate the incidence of PrVI in *Prunus* orchards.

## 2. Results

### 2.1. Analysis of the HTS Data

**Figure 1:**
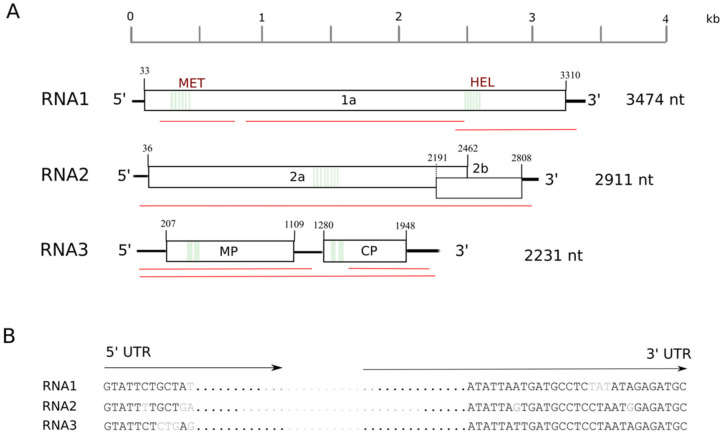
Genomic structure of prunus virus I (PrVI). (**A**) Schematic representation of PrVI genome organization. White boxes show positions of different open reading frames (ORFs), black lines depict untranslated genomic regions (UTRs) and green, vertical lines indicate conserved motifs in ilarviruses’ genomes. Red lines indicate the locations of contigs. (**B**) Length and conserved nucleotide sequences detected at the 5′ and 3′ termini of the three genomic RNA segments of PrVI. Conserved nucleotides are presented with black capital letters, whereas mismatches are highlighted with grey letters. Abbreviations: MET = methyltransferase, HEL = helicase, MP = movement protein, CP = capsid protein.

A total of 14,001,093 reads were obtained after trimming and quality control of the sequences. A host genome subtraction was deployed using the *Prunus avium* scaffolds, resulting in 3,987,365 reads. The de novo assembly with Trinity’ (v.2.2.0) [5] produced 32,050 contigs, ranging from 201–14,021 nt in length. Subsequent BLASTn/BLASTx analysis of the obtained contigs revealed the presence of sequences corresponding to cherry virus A, prunus virus F, little cherry virus 1, cherry necrotic rusty mottle virus, and the recently discovered cherry virus Turkey [4]. Moreover, 7 contigs, ranging from 729 to 2902 nts (Figure 1, red lines), shared nucleotide similarities with genomic sequences of members of the genus *Ilarvirus* (query coverage, 82.6–100%, average identity, 73.4%). Iterative mapping of the reads to the assembled contigs (Supplementary Figure S1) was applied in an attempt to obtain the complete nucleotide sequences from all three segments. Nevertheless, in silico analysis could neither retrieve the untranslated regions (UTRs) of PrVI, nor a small sequence gap in the 5′ terminal of RNA1 (Supplementary Figure S1). For this reason, the complete genome sequence of PrVI was confirmed by Sanger sequencing and Rapid amplification of cDNA ends (RACE).

### 2.2. Genome Structure and Encoded Proteins of the New Ilarvirus

**Figure 2:**
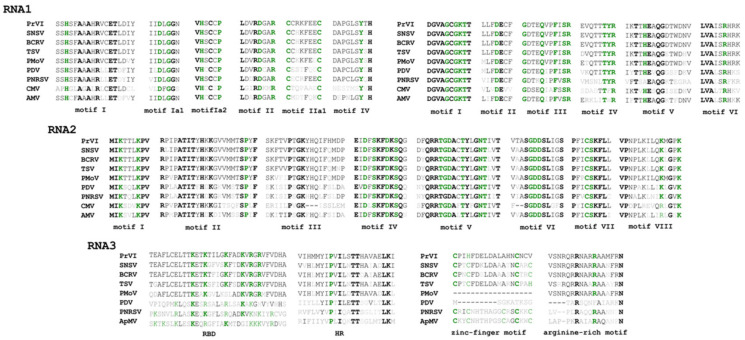
Multiple alignment of the putative amino acid motifs and signatures detected in PrVI and other relative ilarviruses/bromoviruses. Amino acids conserved among all viruses are indicated with green/bold letters, whereas amino acids conserved in all ilarviruses are highlighted with black, bold letters. Grey letters indicate amino acid mismatches with PrVI residues.

The full-length sequences of the three RNA segments of the ilarvirus consist of 3474 nt (RNA1), 2911 nt (RNA2) and 2231 nt (RNA3) (Figure 1A) and they are deposited in the GenBank database under the accession numbers MW579753-5, respectively. Accordingly, RNA1 contains a single ORF1 (3273 nt) encoding a replicase (protein 1a) of 1090 aa, including a methyltransferase (MET) domain (aa location 58–448) and a helicase (HEL) domain (aa location 803–1059) (Figure 1A and Figure 2). RNA2 contains two ORFs (Figure 1A). ORF2a (2427 nt) encodes a putative 809 aa long RNA-dependent-RNA-polymerase protein (RdRp, 2a protein) and includes all the eight conserved motifs (I-VIII) described for the Supergroup III of the RdRps of positive-strand RNA viruses [5] (Figure 2). ORF2b (618 nt) encodes the putative protein 2b (206 aa long), which is unique to ilarvirus subgroups 1 and 2 and is suggested to be involved in viral movement and gene silencing (Pallas et al., 2013; Shimura et al., 2013). Finally, the 5′ and 3′ parts of RNA3 encode the MP (903 nt, 301 aa) and the viral CP (669 nt, 223 aa), respectively (Figure 1A). The 5′ part of the MP protein contains an RNA-binding domain (RBD, aa location 49–80) and a hydrophobic region (HR, aa location 82–103), whereas a zinc-finger (aa location 13–30) and an arginine-rich (aa location 34–50) motif were identified at the 5′ termini of CP (Figure 2).

**Figure 3:**
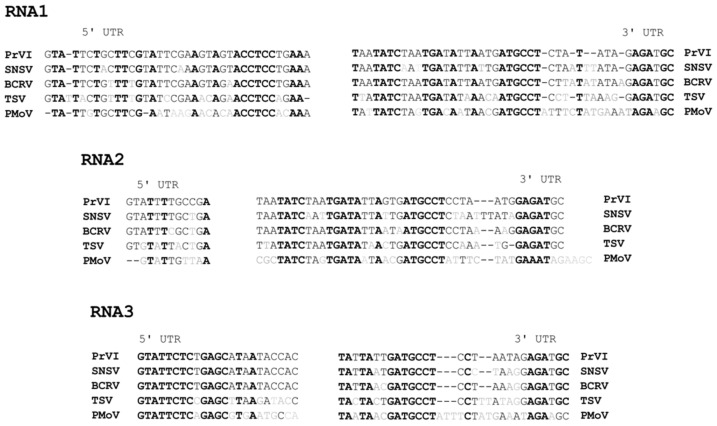
Multiple alignment of the 5′ and 3′ terminal nucleotide sequences of PrVI and other subgroup 1 ilarviruses. Conserved nucleotides are highlighted in bold.

The three genomic RNAs of PrVI start with ‘GTATT’, whereas the terminal nucleotide sequences of all three segments show high sequence similarity (Figure 1B). UTRs share conserved nucleotide residues with closely related ilarviruses, such as strawberry necrotic shock virus (SNSV), blackberry chlorotic ringspot virus (BCRV), tobacco streak virus (TSV), and parietaria mottle virus (PMoV) (Figure 3).

### 2.3. Nucleotide/Amino Acid Comparisons and Phylogenetic Analysis

**Figure 4:**
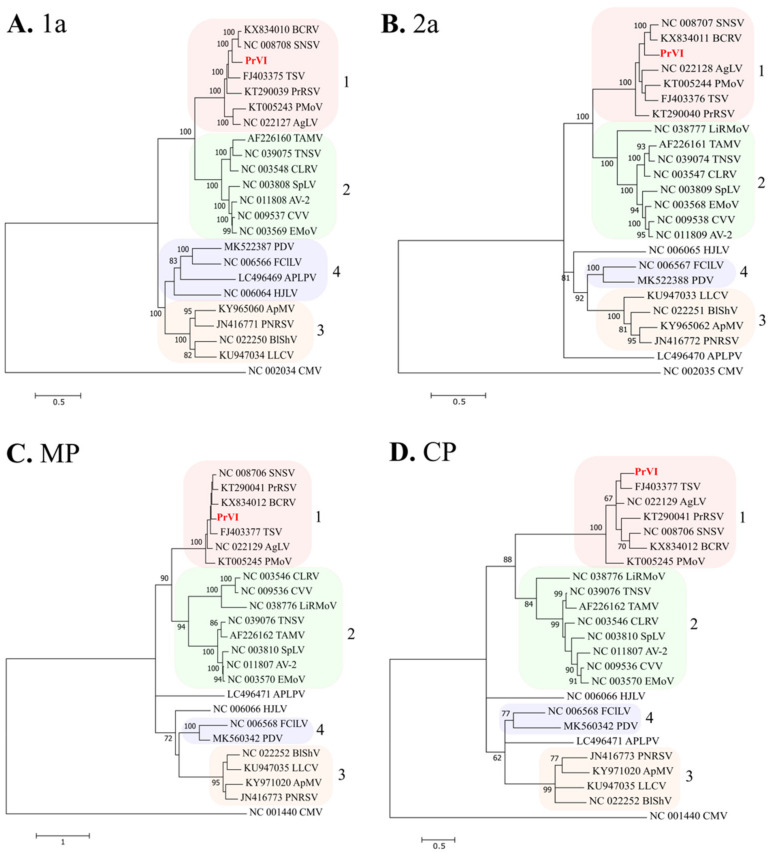
Phylogenetic analysis of the amino acid sequences of (**A**) 1a, (**B**) 2a, (**C**) MP and (**D**) CP of PrVI and other ilarviruses. The trees were constructed using the best fit model for each alignment. The phylogenetic trees were constructed using the Maximum Likelihood method and the statistical significance of branches was evaluated by bootstrap analysis (500 replicates). Bootstrap values above 60% are indicated on the branches. The acronyms are used as follows: ageratum latent virus, AgLV (NC_022127-29); American plum line pattern virus, APLPV (LC496469-71); apple mosaic virus, ApMV (KY965060, KY965062, KY971020); asparagus virus 2, AV-2 (NC_011807-9); blackberry chlorotic ringspot virus, BCRV (KX834010-12); blueberry shock virus, BlShV (NC_022250-52); citrus leaf rugose virus, CLRV (NC_003546-8); citrus variegation virus, CVV (NC_009536-8); elm mottle virus, EMoV (NC_003568-70); fragaria chiloensis latent virus, FClLV (NC_006566-8); humulus japonicas latent virus, HJLV (NC_006064-6); lilac leaf chlorosis virus, LLCV (KU947033-5); lilac ring mottle virus, LiRMoV (NC_038776-7); parietaria mottle virus, PMoV (KT005243-5); privet ringspot virus, PrRSV (KT290039-41); prune dwarf virus, PDV (MK522387-88, MK563042); prunus necrotic ringspot virus, PNRSV (JN416771-3); spinach latent virus, SpLV (NC_003808-10); strawberry necrotic shock virus, SNSV (NC_008706-8); tobacco streak virus, TSV (FJ403375-7); tomato necrotic streak virus, TomNSV (NC_039074-6); tulare apple mosaic virus, TAMV (AF226160-2). Cucumber mosaic virus, CMV (NC_00234-5, NC001440) was used as outgroup. Scale refers to amino acid substitutions per site.

Nucleotide and amino acid comparisons of the new ilarvirus’ ORFs with their cognates of other ilarviruses are illustrated in Supplementary Table S1. The complete nucleotide sequences of all RNA segments share 68.1–71.9% identity with SNSV (accession numbers NC_008706-8) and 66.4–71.6% identity with BCRV (accession numbers KX834010-12). Maximun-likelihood trees based on the aa alignments of the replicase (1a), RdRp (2a), MP and CP proteins of 22 ilarviruses showed that the virus is clustered in all cases in subgroup 1 with TSV, PMoV, SNSV, and BCRV (Figure 4).

### 2.4. Presence of PrVI in Prunus spp.

A total of 138 samples (68 from sweet cherry trees, 58 from peaches and 12 from plums) were also tested for the presence of PrVI by RT-PCR and using specific primers PrVI-CP-F/PrVI-CP-R, designed in this study. RT-PCR revealed the presence of PrVI in four sweet cherry samples collected in 2009, 2013, 2014, and 2019, and in one peach sample collected in 2019 (Table 1). The presence of PrVI in all 5 samples was verified by Sanger sequencing of the obtained amplicons and Greek isolates shared 97.7–98.7% nt identity in nucleotide level. These sequences were accessioned under the numbers presented in Table 1.

## 3. Discussion

The genus *Ilarvirus* (family Bromoviridae) is composed of 22 permanent virus species, according to the ICTV taxonomy, whilst five tentative ilarviruses await official ICTV designations [6]. From a phylogenetic point of view, ilarviruses are classified into four major subgroups: 1, 2, 3, and 4, whereas APLPV and humulus japonicus latent virus show no close relationships to the other subgroups [1]. Nevertheless, all the members of the genus share a few common features in their genome organization. The RNA1 is monocistronic, coding for their viral replicase (1a) and featuring a MET and a HEL domain. In subgroup 1 and subgroup 2 ilarviruses, RNA2 is bicistronic; apart from the viral polymerase (2a), they may encode a smaller protein (2b), through subgenomic RNA [1]. Finally, RNA3 codes for the MP (proximal ORF) and the CP (distal ORF).

In this study, we characterized the complete nucleotide sequence of a novel ilarvirus from a sweet cherry tree, collected from Imathia region. Collective data from in silico analysis revealed that PrVI shares a similar genome organization with ilarviruses and harbors several motifs that are typical of the majority of members in the genus or even in the Bromoviridae family. Phylogenetically, PrVI is closely related to SNSV, BCRV, TSV, PMoV, privet ringspot virus, and ageratum latent virus, all members of the subgroup 1 of ilarviruses (Figure 4). Therefore, PrVI consists the first ilarvirus from subgroup 1 that infects *Prunus* spp.

Furthermore, PrVI UTRs showed high nucleotide similarities with those of their cognate ilarviruses from subgroup I. A series of studies have shown that alfalfa mosaic virus (AMV) and ilarviruses require the interaction of their own CP with the 3′UTRs of their viral RNAs to initiate replication and establish infection [7,8] and this function was termed “genome activation” [9]. Interestingly, a highly conserved R residue located in the CP of AMV, citrus variegation virus and TSV, which is also present in PrVI (Figure 3) and two R residues in PNRSV, were shown to be crucial for this phenomenon to occur [10,11]. Altogether, the conserved nature of these residues in the CP of AMV and ilarviruses along with the nucleotide conservation in their 3′UTRs could facilitate this process.

A small-scale survey was conducted in Imathia region, which consists a major area of stone fruit tree cultivation in Greece. The purpose of this survey was to investigate the presence of PrVI in sweet cherry and other stone fruit trees. In this context, in 2019–2020, we collected samples from sweet cherry, peach and plum orchards as so to identify potentially hosts for PrVI and we used plant material from 2009, 2013–2014 from the collection of the Plant Pathology Lab (AUTH). PrVI was identified in a low incidence of 3.6% (4/138) in sweet cherries. Notably, PrVI was also detected in one peach sample. Nonetheless, further experimental data are needed to address whether peach consists another host for PrVI and, possibly, plum, as we only tested a small number of plum samples. Moreover, the virus was identified in samples collected during 2009, 2014 and 2019 thus indicating that PrVI is infecting trees in the area for over a decade.

It is worth mentioning that the *Prunus* samples that were found positive to PrVI, did not exhibit any obvious symptoms of viral infection. As fulfilling Koch’s postulates faces several limitations [12,13], especially in the case of viruses infecting perennial hosts, the implementation of alternative strategies to associate viruses with putative disease development, such as the simplified hierarchical approach proposed by Fox [12], could provide a wealth of information on PrVI pathogenicity and this topic awaits future research.

## 4. Materials and Methods

### 4.1. High throughput Sequencing and Bioinformatics Analysis

Total RNA was extracted from leaves of the sweet cherry sample c18 using the TRIzol reagent (Invitrogen, Carlsbad, CA, USA) and it was subjected to HTS analysis on an Illumina Hi-seq 4000 platform (Novogene Co., Tianjin, China). Paired-end reads were trimmed using PrinSeq and quality was checked with FastQC (Babraham Bioinformatics, Cambridge, UK). Prior to de novo assembly, the host genome sequences (https://plants.ensembl.org/Prunus_avium/Info/Index, accessed on 3 September 2020) were removed using the ‘Bowtie2′ mapper incorporated in Geneious Prime^®^ 2019.1.1. (https://www.geneious.com, accessed on 3 September 2020). Then, reads were assembled into contigs using the de novo assembler ‘Trinity’ (v.2.2.0) [14] with default parameter settings. BLASTn/BLASTx analysis of the contigs were performed against local and online databases. Iterative mapping of the reads to the assembled contigs was applied with ‘Bowtie2′ implemented in Geneious Prime^®^ 2019.1.1.

### 4.2. Sanger Sequencing of the Novel Ilarvirus Genome

In order to validate the correctness of the de novo assembly, Sanger sequencing of overlapping amplicons with virus-specific primers (Supplementary Table S2) and RACE assays were implemented on new total RNA extracts obtained from the infected tree. For 3′-RACE, a poly-A tail was added to the total RNA using Escherichia coli poly(A) polymerase (NEB, Ipswich, MA, USA) according to the manufacturer’s instructions. Then, the 3′ ends of the three RNA segments were determined via RT-PCR and nested PCR using virus-specific primers and an oligo(d)T primer with an anchor sequence (Supplementary Table S3). The 5′ ends of the viral genome were determined using the commercial ‘5′/3′ RACE Kit, 2nd Generation’ (Roche Diagnostics GmbH, Mannheim, Germany). All amplification products were directly sequenced at Genewiz (Leipzig, Germany) and sequences of overlapping fragments were assembled using the Geneious Prime^®^ 2019.1.1.

### 4.3. Sequence Analysis and Phylogenetic Reconstructions

Full-length viral genome sequences were submitted to ORF finder [15] to determine the genomic organization and the predicted amino acid (aa) sequences of the gene products. MAFFT v.7.450 was used as the multiple alignment program for nucleotide and amino acid sequences. For the phylogenetic comparisons of complete coding regions presented in all ilaviruses, i.e., 1a, 2a, MP, and CP, 22 established ilarvirus species were used. Maximum likelihood (ML) trees were constructed using MEGA 7 [16], applying appropriate, amino acid substitution models calculated by ModelTest-NG [17]. The reliability of the phylogenetic hypothesis was evaluated using nonparametric bootstrap analysis (NPB).

### 4.4. Mini-Scale Survey for the Presence of PrVI in Greek Orchards

A small-scale survey was conducted in 2019-2020 in Imathia region, with a view to investigate the presence of PrVI in *Prunus* spp., such as sweet cherry, peach and plum. Moreover, plant material was also used from the collection of the Laboratory of Plant Pathology (Aristotle University of Thessaloniki) from previous years, including Imathia and Arta counties (Table 1). A total of 138 samples were tested and processed to total RNA extraction according to a CTAB-based protocol described by Gambino and colleagues [18]. One-tube RT PCR was deployed using the primer set CP-F (5′-AACGATGCTATCACACTGAAGAC-3′)/CP-R (5′-CTCTGGTGGCAGCAAAGGC-3′) amplifying a 351 bp fragment of the CP gene. In detail, 1 μg of total RNA was used as a template in a reaction mixture containing 10mM Tris-HCl (pH 8.9), 50 mM KCl, 2.5 mM MgCl_2_, 0.2 mM each dNTP, 0.4 μM of the primer set CP-F/CP-R, 3 U MMLV (Invitrogen) 1.5 U GRS HotStart Taq DNA polymerase (GRiSP Research Solutions) and DEPC-treated water to a final volume of 25 μL. The cycling conditions were 45 °C for 30 min; 94 °C for 5 min, 40 cycles of 94 °C for 30 s, 57 °C for 30 s, and 72 °C for 20 s; and a final extension at 72 °C for 2 min. Amplicons derived from the RT-PCR assays were directly submitted for Sanger sequencing at Genewiz (Leipzig, Germany).

## Figures and Tables

**Table 1:** Presence of PrVI in Greek *Prunus* spp. orchards.

*Prunus* spp.	Collection Date	Geographic County	Total Number of Samples	Number of Samples Positive to PrVI	Accession Numbers
*P. avium*	2009	Imathia	12	1	MW591551
	2014				MW591550
		Imathia	10	2	
					MW591552
	2019	Imathia	31	1	MW591554
	2020	Imathia	15	0	- ^1^
*P. persica*	2013	Arta	12	0	-
	2014	Imathia	11	0	-
	2019	Imathia	6	1	MW591553
	2020	Imathia	29	0	-
*P. domestica*	2020	Imathia	12	0	-
SUM			138	5	

^1^ Not applicable.

## Supplementary Materials

The following are available online at https://www.mdpi.com/2223-7747/10/3/514/s1, Table S1: Nucleotide and amino acid sequence identities between predicted proteins encoded from the genome of prunus virus I (PrVI) and their homologs from other ilarviruses; Table S2: Primers used for complete genome sequencing of PrVI from sweet cherry; Table S3: Primer sequences used for RACE; Figure S1: Iterative mapping of the reads to the assembled contigs corresponding to all three RNA segments of PrVI.
