# Supplementary material for: Identification and Sequence Analysis of a Novel Ilarvirus Infecting Sweet Cherry
Source: Plants (Basel). 2021 Mar 10;10(3):514. doi: 10.3390/plants10030514 (PMC8000932; doi:10.3390/plants10030514)
Supplement: Supplementary file 1 [file plants-10-00514-s001.pdf]

Supplementary material

Table S1. Nucleotide and amino acid sequence identities between predicted proteins encoded from the genome of prunus virus I (PrVI) and their homologs from other ilarviruses. Ilarviruses that are closely related to PrVI were highlighted in grey shadow.

Virus acronym	RNA1	ORF1		RNA2	ORF2a		ORF2b		RNA3	ORF3 (MP)		ORF4 (CP)	
	nt	nt	aa	nt	nt	aa	nt	aa	nt	nt	aa	nt	aa
**AgLV**	64.6	68.1	71.4	67.0	67.6	69.0	62.7	56.8	62.9	63.3	68.4	66.8	60.9
**APLPV**	39.1	41.4	31.0	35.7	38.3	30.8	-	-	27.6	31.8	19.5	31.9	11.5
**ApMV**	41.1	43.6	35.5	32.5	36.6	31.7	-	-	27.0	32.1	16.6	25.3	11.9
**AV-2**	47.8	49.9	45.8	42.4	44.7	38.0	30.6	19.2	33.2	38.3	25.8	33.3	15.5
**BCRV**	69.4	72.3	80.5	70.7	70.9	73.2	69.6	64.9	64.1	69.1	71.4	59.0	53.4
**BlShV**	42.0	43.9	35.5	34.5	37.2	33.7	-	-	27.3	31.9	18.8	28.2	11.7
**CLRV**	47.0	50.4	43.7	42.2	42.5	36.7	32.2	15.6	31.5	35.6	23.4	33.6	15.7
**CVV**	47.8	49.8	44.0	41.6	44.3	37.9	32.2	20.6	32.2	34.6	21.9	35.6	17.0
**EMoV**	48.2	51.0	44.5	41.8	43.6	38.0	31.4	14.0	34.0	38.6	25.8	34.9	16.3
**FClLV**	41.8	44.5	34.7	34.5	36.9	33.6	-	-	27.7	35.3	21.6	27.6	14.4
**HJLV**	40.5	42.6	33.5	33.8	35.2	28.0	-	-	28.3	34.6	19.1	28.6	13.5
**LLCV**	39.7	42.6	34.7	34.5	36.9	32.0	-	-	25.9	30.7	16.6	27.3	12.0

**LiRMoV**	-	-	-	40.9	43.0	37.2	29.5	17.1	30.0	35.0	23.1	30.6	16.9
**PMoV**	62.6	66.0	69.2	64.6	65.5	67.6	58.9	51.9	53.4	61.7	65.7	54.9	47.2
**PrRSV**	66.5	69.3	73.7	64.9	64.1	65.8	59.2	54.4	59.3	65.6	72.7	57.7	53.0
**PDV**	41.3	43.4	33.4	36.0	38.3	33.5	-	-	28.4	36.3	22.4	26.2	11.1
**PNRSV**	39.9	42.3	34.2	33.9	37.7	32.3	-	-	27.0	32.3	17.9	28.9	13.1
**SpLV**	47.5	50.4	43.7	40.7	43.7	37.3	33.6	18.4	33.6	38.7	25.7	36.8	17.8
**SNSV**	70.3	73.1	79.8	70.5	70.9	71.5	66.7	58.2	66.0	69.2	74.1	62.0	62.0
**TSV**	65.9	69.6	75.0	65.7	65.9	66.7	58.0	54.7	63.6	64.8	73.0	65.1	59.8
**TomNSV**	47.0	50.0	44.0	42.1	42.0	37.1	31.1	15.2	33.2	37.6	24.2	33.6	15.3
**TAMV**	45.9	48.8	41.3	42.2	43.0	37.5	31.8	19.6	33.7	37.6	22.5	32.4	13.2

Table S2. Primers used for complete genome sequencing of PrVI from sweet cherry.

Name of primer	RNA segment	Primer sequence (5′→3′)	Amplicon length (bp)	Ta (°C)*
s1-F1	RNA1	TAGGGCGGACGATACAACAAAC	760	63
s1-R1		GACGTGCTGTCGAAATATATGACT		
s1-F2		ATATTGCTATTGAAACCGTAGCAC	920	60
s1-R2		GTTTACGTATGTTTAGAAAGAAACAA		
s1-F3		CAATTCATTTGGATGAGTATGTACCA	1,002	60
s1-R3		GGATGGTTACGTGACTCTCC		
s1-F4		TGTTACGAACCCTTATGCCG	992	62
s1-R4		GGCTGATAAGGACGTCCTTA		
s1-F5		CCGCATGCAACACCAAAGAG	596	63
s1-R5		CTAAACATGGTTGTTTAGTGCTGTAT		
s2-F1	RNA2	ACGTGAAAGAAAACCTCATTCTATTT	1,060	62
s2-R1		CGAGATTGGCAAAGTTGTTACC		
s2-F2		CCTGGACCTGTCAGTCTTTAATG	979	63
s2-R2		CGAGACGAAGTTTCCACACA		
s2-F3		ATGCCCATGAAGTAAGTCACATTC	984	63
s2-R3		TTAACTTCGAAGTTTCTGAATTTCCC		
s2-F4		AAAGTGTTCCGATGCCAAACC	583	61
s2-R4		CTCGTATGTATCTCTACACGAGT		
s3-F1	RNA3	CGAATCGGACGATTTCCAACT	989	62
s3-R1		GTTGGTATTAACCTTTTGGAACCTA		
s3-F2		AGACATTCACGAGAACAATGATAAG	990	62
s3-R2		GAACACATATCGAAGCCGAGA		
s3-F3		AGACCACTGATACCAACGAT	614	61
s3-R3		AAGGTGAGGCCTTTGGGC		

*Annealing temperatures were calculated at http://tmcalculator.neb.com/#!/main.

Table S3. Primer sequences used for RACE.

RNA segment	RACE	Name of primer	Primer sequence (5′→3′)	Ta (°C)
RNA1	5′ first PCR	5-RACE-R1	GGAGGTGTCAACTTGAGATTTC	57
		AAP[1]	GGCCACGCGTCGACTAGTACGGGIIGGGIIGGGIIG	
	5′ nested PCR	5-RACE-R1n	ACTCTCTCAATGCTACCTCACC	61
		AUAP[1]	GGCCACGCGTCGACTAGTAC	
	3′ first PCR	3-RACE-F1	GTATTTTACCGTTGCACCTCATG	57
		28V[2]	CACGGATCCCGGGTTTTTTTTTTTTTTTTTTV	
	3′ nested PCR	3-RACE-F1n	ACAACGATGATCAGATAGTAAAGTGT	61
		28V	CACGGATCCCGGGTTTTTTTTTTTTTTTTTTV	
RNA2	5′ first PCR	5-RACE-R2	TGAGTAGAACGCCCATCATC	57
		AAP	GGCCACGCGTCGACTAGTACGGGIIGGGIIGGGIIG	
	5′ nested PCR	5-RACE-R2n	CGTCCAACGACCATGCGAAATG	61
		AUAP	GGCCACGCGTCGACTAGTAC	
	3′ first PCR	3-RACE-F2	GTTGGTTACAGACTTTGAAGT	57
		28V	CACGGATCCCGGGTTTTTTTTTTTTTTTTTTV	
	3′ nested PCR	3-RACE-F2n	TGATTTACCGAAGATTCCCAATATAG	61
		28V	CACGGATCCCGGGTTTTTTTTTTTTTTTTTTV	
RNA3	5′ first PCR	5-RACE-R3	ACCACTTAACGCATCTGAAAC	57
		AAP	GGCCACGCGTCGACTAGTACGGGIIGGGIIGGGIIG	
	5′ nested PCR	5-RACE-R3n	AAAGGTGAGAGCACGATAGGTAG	61
		AUAP	GGCCACGCGTCGACTAGTAC	
	3′ first PCR	3-RACE-F3	GTGTGAGATGCGGATTCAG	57
		28V	CACGGATCCCGGGTTTTTTTTTTTTTTTTTTV	
	3′ nested PCR	3-RACE-F3n	TGTAGATCAGTGTATTTGGAGATGTC	61
		28V	CACGGATCCCGGGTTTTTTTTTTTTTTTTTTV	

[1] Alves-Freitas et al. (2019), [2] Gibbs and Mackenzie (1996)

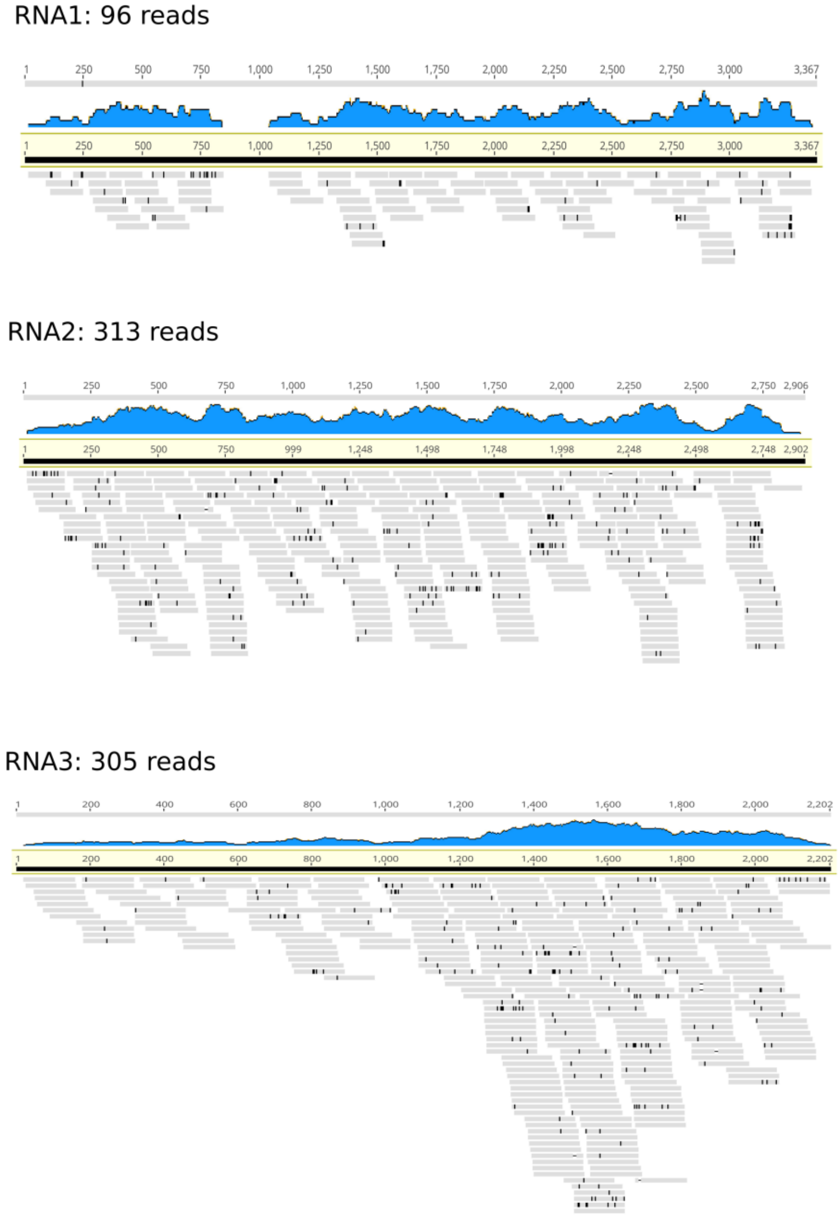

Figure S1. Iterative mapping of the reads to the assembled contigs corresponding to all three RNA segments of PrVI.

References

1. Alves-Freitas, D.M.T.; Pinheiro-Lima, B.; Faria, J.C.; Lacorte, C.; Ribeiro, S.G.; Melo, F.L. Double-stranded RNA high-throughput sequencing reveals a new cytorhabdovirus in a Bean golden mosaic virus-resistant common bean transgenic line. *Viruses* **2019**, 11:90. https://doi.org/10.3390/v11010090
2. Gibbs, A.; Mackenzie, A. A primer pair for amplifying part of the genome of all potyvirids by RT-PCR. *J. Virol. Methods* **1996**, 63:9–16. https://doi.org/10.1016/S0166-0934(96)02103-9
